# Supplementary material for: Paranoia as a deficit in non-social belief updating
Source: eLife. 2020 May 26;9:e56345. doi: 10.7554/eLife.56345 (PMC7326495; doi:10.7554/eLife.56345)
Supplement: Transparent reporting form [file elife-56345-transrepform.docx]

***eLife’s* transparent reporting form**

We encourage authors to provide detailed information *within their submission* to facilitate the interpretation and replication of experiments. Authors can upload supporting documentation to indicate the use of appropriate reporting guidelines for health-related research (see [EQUATOR Network](http://www.equator-network.org/%20)), life science research (see the [BioSharing Information Resource](https://biosharing.org/)), or the [ARRIVE guidelines](http://www.plosbiology.org/article/info:doi/10.1371/journal.pbio.1000412) for reporting work involving animal research. Where applicable, authors should refer to any relevant reporting standards documents in this form.

If you have any questions, please consult our Journal Policies and/or contact us: [editorial@elifesciences.org](mailto:editorial@elifesciences.org).

**Sample-size estimation**

- You should state whether an appropriate sample size was computed when the study was being designed
- You should state the statistical method of sample size computation and any required assumptions
- If no explicit power analysis was used, you should describe how you decided what sample (replicate) size (number) to use

Please outline where this information can be found within the submission (e.g., sections or figure legends), or explain why this information doesn’t apply to your submission:

We did not compute formal power calculations before gathering our data. Based on effects present in the in-laboratory pilot and our previous work (e.g., Powers, Mathys, and Corlett, Science, 2017), we recruited participants – distributing them among task conditions in our online study via restricted randomization running in batches of 9 participants or less to ensure that we reached >15 participants with high paranoia per task version. Effect sizes have been computed and reported for every statistical comparison reported (described in the Results section and throughout the manuscript).

**Replicates**

- You should report how often each experiment was performed
- You should include a definition of biological versus technical replication
- The data obtained should be provided and sufficient information should be provided to indicate the number of independent biological and/or technical replicates
- If you encountered any outliers, you should describe how these were handled
- Criteria for exclusion/inclusion of data should be clearly stated
- High-throughput sequence data should be uploaded before submission, with a private link for reviewers provided (these are available from both GEO and ArrayExpress)

Please outline where this information can be found within the submission (e.g., sections or figure legends), or explain why this information doesn’t apply to your submission:

Participants for the in-laboratory study were recruited through clinician referrals and public fliers posted around the community (New Haven, CT, USA). Self-selection bias is likely present and to some extent ineradicable due to the inherent mistrust of paranoid individuals. To make our study more accessible to individuals with debilitating paranoia, social anxiety, and other conditions, we performed an online study in which participants could assume relative anonymity (i.e., identifying information limited to worker ID number and IP address). These participants were recruited through a “Human Intelligence Task” (HIT) advertisement in the Amazon Mechanical Turk online marketplace.

We collected data for the in-laboratory experiment from January, 2017 through July, 2017. We collected MTurk data in small batches (up to 9 participants per batch) in December, 2017 and from May through September, 2018.

Experiment 2 entails conceptual replication of Experiment 1. Experiment 3, and the cluster analyses are consistent with conceptual replication of Experiments 1, 2, and 3 across species.

Data exclusion: In laboratory: Two participants were excluded, one 'healthy control' with suspected substance use (pre-determined exclusion criterion), and one who completed the behavioral task but not the questionnaires (insufficient data). Online: In response to reports of virtual private server (VPS) use to circumvent MTurk location requirements and/or submit multiple, poor quality responses, we implemented a policy of excluding suspected VPS and server farm users by checking IP addresses and the quality of answers to open-ended, free response questions. We identified and excluded 19 suspected submissions with IP addresses located outside of the United States or associated with known server farms and nearly identical, nonsensical free responses answers. To identify and exclude potential bots, we required participants to manually enter their MTurk ID number into a free response question. Four participants were excluded for failure to do so. Two additional subjects were excluded for declining entire sections of the questionnaires (insufficient data). We established the anti-bot policy before beginning the experiment. The remaining exclusion criteria were implemented after initiating data collection. Described beginning line 486 in the manuscript.

**Statistical reporting**

- Statistical analysis methods should be described and justified
- Raw data should be presented in figures whenever informative to do so (typically when N per group is less than 10)
- For each experiment, you should identify the statistical tests used, exact values of N, definitions of center, methods of multiple test correction, and dispersion and precision measures (e.g., mean, median, SD, SEM, confidence intervals; and, for the major substantive results, a measure of effect size (e.g., Pearson's r, Cohen's d)
- Report exact p-values wherever possible alongside the summary statistics and 95% confidence intervals. These should be reported for all key questions and not only when the p-value is less than 0.05.

Please outline where this information can be found within the submission (e.g., sections or figure legends), or explain why this information doesn’t apply to your submission:

- Statistical analyses are described in the Methods section (including appropriate corrections for multiple statistical comparisons (beginning line 279).
- All requested statistical details are provided for every analysis described throughout the manuscript.
- Raw data are presented in all figures.

(For large datasets, or papers with a very large number of statistical tests, you may upload a single table file with tests, Ns, etc., with reference to sections in the manuscript.)

**Group allocation**

- Indicate how samples were allocated into experimental groups (in the case of clinical studies, please specify allocation to treatment method); if randomization was used, please also state if restricted randomization was applied
- Indicate if masking was used during group allocation, data collection and/or data analysis

Please outline where this information can be found within the submission (e.g., sections or figure legends), or explain why this information doesn’t apply to your submission:

The in-laboratory experiment recruited from public fliers and clinician referrals. The online experiment used a stratified random sample. Based on effects present in the in laboratory pilot and our previous work (e.g., Powers, Mathys, and Corlett, Science, 2017), we recruited participants until we reached >15 participants with high paranoia per task version (described line 536).

No masking was necessary for these experiments.

**Additional data files (“source data”)**

- We encourage you to upload relevant additional data files, such as numerical data that are represented as a graph in a figure, or as a summary table
- Where provided, these should be in the most useful format, and they can be uploaded as “Source data” files linked to a main figure or table
- Include model definition files including the full list of parameters used
- Include code used for data analysis (e.g., R, MatLab)
- Avoid stating that data files are “available upon request”

Please indicate the figures or tables for which source data files have been provided:

All data and model analysis code are available on ModelDB78 (http://modeldb.yale.edu/258631) with accession code p2c8q74m.
